# Impact of Using Cow’s Milk Formula During the First Three Postnatal Days and Other Etiological Factors on the Development of Cow’s Milk Protein Allergy

**DOI:** 10.3390/jcm14248664

**Published:** 2025-12-07

**Authors:** Halil Sağır, Yeliz Çağan Appak, Betül Aksoy, Sinem Kahveci, Şenay Onbaşı Karabağ, Özlem Gülpınar Aydın, Serenay Alaca, Maşallah Baran

**Affiliations:** 1 Department of Pediatric Gastroenterology, Hepatology and Nutrition, Faculty of Medicine, Izmir Katip Celebi University, Izmir City Hospital, 35540 Izmir, Türkiye; yelizcagan@yahoo.com (Y.Ç.A.); drbetulaksoy@gmail.com (B.A.); drserenaycetinoglu@gmail.com (S.A.); mbaran2509@gmail.com (M.B.); 2 Department of Pediatric Gastroenterology, Hepatology and Nutrition, Izmir City Hospital, 35540 Izmir, Türkiye; dr_skahveci@hotmail.com (S.K.); senayonbasikarabag@gmail.com (Ş.O.K.); ozlem_gulpinar@hotmail.com (Ö.G.A.)

**Keywords:** cow’s milk protein allergy, breastfeeding, cow’s milk formula, cesarean delivery

## Abstract

**Background/Objectives:** Early cow’s milk formula (CMF) exposure in the neonatal period has been proposed as a risk factor for cow’s milk protein allergy (CMPA), although evidence remains inconsistent. This study evaluated the association between CMF use within the first 72 h and later CMPA. **Methods:** This retrospective study included 106 CMPA infants and 106 controls. CMPA was diagnosed according to ESPGHAN 2024 guidelines using clinical assessment, a 2–4-week elimination diet, and a supervised oral food challenge. Demographic and feeding data were collected, and logistic regression identified independent predictors. **Results:** Early CMF exposure was more common in the CMPA group (76% vs. 59%; *p* = 0.006). Multivariate analysis confirmed early CMF exposure as an independent risk factor (aOR: 2.35; 95% CI: 1.18–4.68). A subgroup with CMF intake limited to the first 72 h followed by exclusive breastfeeding was markedly overrepresented among CMPA cases (47% vs. 12%; *p* < 0.001). Early CMF exposure did not significantly impact IgE versus non-IgE phenotype distribution. Family history of atopy also remained a strong predictor. **Conclusions:** Early CMF exposure during the first 72 h and family history of atopy were key risk factors for CMPA. Supporting breastfeeding initiation and avoiding unnecessary early CMF supplementation may help reduce CMPA incidence.

## 1. Introduction

The World Health Organization (WHO) recommends exclusive breastfeeding during the first six months of life and initiating breastfeeding within the first half hour after birth [1]. Breast milk provides numerous immunological and protective factors that safeguard neonates whose intestinal defense mechanisms are not yet fully developed [2]. Failure to initiate breastfeeding immediately after birth, cesarean delivery, and prematurity are among the major factors contributing to delayed lactogenesis [3], which in turn increases the likelihood of cow’s milk formula (CMF) use during the early postnatal period.

The gut microbiota begins to develop during the neonatal period and plays a crucial role in establishing immune tolerance and shaping a functional immune system [4]. This early microbial colonization is subsequently influenced by environmental exposures. Considering the critical immunological and intestinal benefits of colostrum during the first three days of life, CMF introduction during this period may reduce colostrum intake and negatively influence these physiological processes [5].

In the early postnatal period, the intestinal barrier remains functionally immature, and immune responses are largely Th2-biased [6]. The incomplete maturation of the gastrointestinal tract may allow IgE-binding epitopes to retain their native three-dimensional configuration, thereby increasing the likelihood of allergenic sensitization in susceptible infants [7]. Exposure to cow’s milk proteins during this critical window before the establishment of breastfeeding and gut microbial homeostasis may therefore disrupt immune tolerance and predispose infants to cow’s milk protein allergy (CMPA) [4,5].

Food allergy is defined as an abnormal immune response to dietary proteins and has become an increasingly recognized global health problem [8]. A meta-analysis on the prevalence of food allergies identified cow’s milk, egg, fish, and peanuts as the most common allergens [9].

Epidemiological studies indicate that the prevalence of CMPA has increased, likely due to complex interactions among environmental, lifestyle, and dietary factors [10]. The reported incidence varies widely across populations, and overestimation may occur due to misdiagnosis [10]. In a large cohort study involving 12,049 infants, CMPA was suspected in 355 cases but confirmed by oral food challenge in only five, corresponding to a prevalence of 0.54% [10]. Another study reported that the prevalence could reach as high as 3.8% [11]. While the risk of CMPA is estimated at 0.5% among exclusively breastfed infants, it increases to 2–3% among those fed with CMF [12].

CMPA can be classified into three phenotypes: IgE-mediated, non-IgE-mediated, and mixed-type reactions. According to EuroPrevall data, the prevalence of non-IgE-mediated CMPA is approximately 1% [13]. Non-IgE-mediated CMPA encompasses a wide clinical spectrum, ranging from mild rectal bleeding to severe vomiting and even sepsis-like presentations, as observed in food protein-induced enterocolitis syndrome. In the United Kingdom, most infants presenting with suspected CMPA were diagnosed with mild to moderate non-IgE-mediated disease [14].

Additional risk factors for CMPA development include prematurity, cesarean delivery, reduced breastfeeding, and a maternal history of allergy [13]. Cesarean delivery has been identified as a potential risk factor for CMPA, possibly due to altered microbial colonization and delayed immune adaptation [15]. Although the World Health Organization recommends that cesarean section rates should not exceed 10–15% at the population level, a recent Turkish cohort study analyzing births over a five-year period reported an alarmingly high cesarean delivery rate of 57% [16].

This period is particularly important because it represents a developmental window that is minimally influenced by environmental exposures, yet the immunological effects of colostrum during these early days remain insufficiently studied and are subject to conflicting views; therefore, identifying the association between early CMF use and CMPA, especially in the context of increasingly prevalent risk factors such as cesarean delivery, may provide critical evidence for developing public health strategies aimed at reducing unnecessary CMF exposure.

Therefore, this study aimed to investigate the association between CMF use during the first three postnatal days and the subsequent development of CMPA, as well as other potential etiological factors.

## 2. Materials and Methods

### 2.1. Study Design and Ethics

This retrospective, single-center study was conducted at the Department of Pediatric Gastroenterology, Hepatology, and Nutrition, İzmir City Hospital, Turkey. The study protocol was approved by the local institutional ethics committee (approval number: 2025/143). Written informed consent was waived due to the retrospective design.

### 2.2. Study Population

Infants diagnosed with CMPA in the pediatric gastroenterology clinic between September 2024 and March 2025 were included in the study. The absence of a single highly sensitive and specific diagnostic test for CMPA makes confirmation challenging and may also lead to unnecessary diagnoses in clinical practice. IgE-mediated CMPA typically presents with acute-onset symptoms occurring within minutes to hours after exposure, such as urticaria or respiratory manifestations, whereas non-IgE-mediated CMPA is characterized by delayed gastrointestinal symptoms appearing more than two hours after ingestion. In functional gastrointestinal disorders such as regurgitation, infantile colic, and functional constipation, premature initiation of elimination diets may contribute to an overdiagnosis of CMPA.

In this study, CMPA was diagnosed in accordance with the 2024 ESPGHAN guidelines using a combination of clinical findings, an elimination diet, and an oral food challenge. Infants presenting with gastrointestinal symptoms, including bloody or mucoid stools, persistent vomiting, or significant constipation and/or urticarial reactions following CMF exposure, underwent a 2–4-week elimination of cow’s milk protein [17]. All infants were followed on an outpatient basis. Those who exhibited clear improvement during the elimination phase subsequently underwent a supervised oral food challenge. The challenge was considered positive when gastrointestinal and/or dermatological symptoms recurred within 48–72 h of reintroduction, consistent with ESPGHAN recommendations for non-IgE-mediated CMPA. Infants with a documented history of anaphylaxis after dairy ingestion were classified as having CMPA without the need for additional testing.

Although early CMF exposure occurred during the first 72 h of life, CMPA was not diagnosed at birth. Instead, the diagnosis was confirmed later in infancy after appropriate clinical follow-up using ESPGHAN-recommended elimination and supervised oral food challenge procedures, ensuring that only clinically verified CMPA cases were included in the study.

To avoid misclassification, infants with gastrointestinal disorders that could mimic CMPA, such as short bowel syndrome, eosinophilic esophagitis, eosinophilic enteropathy, or inflammatory bowel disease, were excluded from the study.

In Turkey, donor human breast milk is not available within the national healthcare system, and none of the participating hospitals had access to donor milk during the study period. Therefore, in all cases where maternal breast milk was insufficient, supplementation was provided exclusively with cow’s milk formula, as donor milk was not an option.

### 2.3. Control Group

The control group consisted of healthy outpatient children referred for routine follow-up. Neither the children nor their mothers had a history of food elimination diets, and no medication other than standard vitamin D and iron supplementation was used. Children with a history of hospitalization, chronic medical conditions, or additional medication use were excluded.

Cases and controls were not individually matched for age or sex; instead, age and sex distributions were compared during the analysis. The age difference between groups reflects the typical early onset of CMPA, which generally presents within the first 6 months of life, leading to naturally younger ages among CMPA cases compared with controls.

### 2.4. Data Collection

Data were obtained from patient files electronically organized by our research team. Incomplete or missing data were excluded from the analysis. The following variables were recorded: age, sex, gestational age, mode of delivery, history of neonatal intensive care unit (NICU) admission, cow’s milk formula (CMF) exposure within the first 72 h of life (including single exposures), family history of atopy, antibiotic exposure, absolute eosinophil counts, and serum milk-specific immunoglobulin E (IgE) levels. Infants born before 37 weeks of gestation were classified as preterm. Hypereosinophilia was defined as an absolute eosinophil count >500 cells/µL. Milk-specific IgE results were considered positive when ≥0.35 kU/L.

CMF exposure was defined exclusively as the consumption of standard cow’s milk-based infant formula. Minimal exposure to cow’s milk protein through human milk fortifiers (HMF) was not classified as CMF exposure. HMF use was uncommon in our outpatient cohort, and detailed information regarding HMF administration was not consistently available in the retrospective records; therefore, only infants with documented intake of standard CMF were included in the CMF-exposed group.

Although detailed records on maternal dietary habits during pregnancy were not available, none of the mothers reported adhering to a special diet or undertaking any elimination diet during the gestational period.

### 2.5. Statistical Analysis

Sample size was determined using an a priori power analysis with G*Power 3.1 software. With an effect size of w = 0.50, a type I error rate of 0.05, and a power of 95%, the minimum required sample size was 105 subjects per group.

Statistical analyses were performed using IBM SPSS Statistics version 26.0 (IBM Corp., Armonk, NY, USA). Descriptive statistics are expressed as numbers (n), percentages (%), means ± standard deviations (SDs), and medians with minimum and maximum values, as appropriate. The normality of continuous variables was assessed using the Shapiro–Wilk test. Normally distributed variables were compared using the independent-samples *t*-test, while non-normally distributed variables were compared using the Mann–Whitney U test. Categorical variables were analyzed using the chi-square test or Fisher’s exact test when applicable.

Binary logistic regression analysis was performed to identify independent predictors of CMPA. All clinically relevant variables, including early CMF exposure, mode of delivery, prematurity, NICU admission, neonatal antibiotic exposure, and family history of atopy, were assessed in univariate analyses. Variables meeting the predefined threshold of *p* < 0.10 (early CMF exposure and family history of atopy) were subsequently entered into the multivariate logistic regression model.

### 2.6. Transparency and Data Availability

To ensure transparency and reproducibility, all statistical analyses were conducted according to standardized procedures. De-identified data supporting the results of this study are available from the corresponding author upon reasonable request.

## 3. Results

### 3.1. Study Population and General Characteristics

A total of 212 infants were included in the study, comprising 106 with cow’s milk protein allergy (CMPA) and 106 controls. The two groups were compared in terms of sex, gestational age, mode of delivery, timing of birth (term or preterm), NICU admission, and peripheral hypereosinophilia proportion of males was 51% in the CMPA group and 52% in controls, with no significant difference in sex distribution (*p* = 0.500). Most infants were born at term (CMPA: 84%, control: 83%; *p* = 0.500). Cesarean section rates were 76% in the CMPA group and 69% in the control group, without a statistically significant difference (*p* = 0.140). Admission to the neonatal intensive care unit (NICU) was reported in 25% of CMPA patients and 26% of controls (*p* = 0.437). Demographic characteristics of both groups are summarized in Table 1, confirming the overall comparability of the study population.

### 3.2. Clinical Characteristics

The most common presenting symptoms in the CMPA group were bloody stools (42%), mucoid stools (29%), vomiting (8%), constipation (8%), skin manifestations (8%), diarrhea (3%), and anaphylaxis (1%). CMPA diagnosis was confirmed at a mean age of 3.6 ± 1.9 months, with a median age of 3 months (range: 1–12 months).

Due to the retrospective nature of the study, the ages of the infants at enrollment differed from the age at diagnosis. At the time of inclusion, infants in the CMPA group had a mean age of 9.4 ± 6.7 months, with a median of 8 months (range: 1–40 months), whereas those in the control group had a mean age of 12.0 ± 8.1 months, with a median of 11 months (range: 2–37 months). A family history of atopy was significantly more frequent among CMPA patients compared with controls (59% vs. 20%, *p* < 0.001; odds ratio [OR]: 6.57; 95% confidence interval [CI]: 3.51–12.19). Hypereosinophilia was observed in 12% of 91 CMPA patients and in 29% of 24 controls (*p* = 0.045). Milk-specific immunoglobulin E (IgE) testing was performed in 43 CMPA patients, of whom 21% had positive results. Demographic and clinical characteristics of the CMPA and control groups are summarized in Table 1.

### 3.3. Risk Factor Analysis

Cow’s milk formula (CMF) exposure during the first 72 h of life was observed in 68% of all infants and was significantly more frequent in the CMPA group compared with controls (76% vs. 59%; *p* = 0.006).

As the study was retrospective, detailed quantitative data on the exact amount of CMF administered were limited. Among infants who received early CMF in the CMPA group, 29 (36%) had only a single formula feeding, compared with two infants in the control group.

In the CMPA group, early exposure to cow’s milk formula (CMF) within the first 72 h of life was identified in 81 children (76%), whereas this rate was 63 children (59%) in the control group. Among children without CMF exposure during the first 72 h, later CMF introduction was recorded in 5 children in the CMPA group and 11 children in the control group.

Breastfeeding continued in most infants who were exposed to CMF. The number of exclusively breastfed children was 20 in the CMPA group and 32 in the control group. Additionally, 22 children (21%) in the CMPA group had no CMF exposure at any time, while 23 children had no exposure specifically during the first 72 h of life. One infant received a single CMF feeding only after the first 72 h period.

Early CMF exposure within the first 72 h was significantly more frequent in the CMPA group compared with exclusively breastfed infants (*p* = 0.006).

Univariate logistic regression analysis demonstrated that early CMF exposure was significantly associated with an increased risk of CMPA (OR: 2.21; 95% CI: 1.22–4.00; *p* = 0.009). A family history of atopy showed an even stronger association with CMPA (OR: 5.93; 95% CI: 3.21–10.97; *p* < 0.001). Cesarean delivery, prematurity, and NICU admission were not significantly associated with CMPA in univariate analyses and therefore did not meet statistical criteria for inclusion based solely on univariate significance.

In the multivariate logistic regression model, early CMF exposure during the first 72 h (adjusted OR: 2.35; 95% CI: 1.18–4.68; *p* = 0.016) and a family history of atopy (adjusted OR: 6.29; 95% CI: 3.31–11.92; *p* < 0.001) remained independent predictors of CMPA. Cesarean delivery, prematurity, and NICU admission did not retain statistical significance after adjustment.

When the pattern of CMF exposure was examined in more detail, a distinct subgroup emerged. Among infants with any CMF exposure, early and limited exposure confined to the first 72 h followed by exclusive breastfeeding was markedly more common in the CMPA group than in controls (40/86 [47%] vs. 9/74 [12%]; *p* < 0.001; OR: 6.42; 95% CI: 2.84–14.53).

Multivariate logistic regression findings are summarized in Table 2, and the comparison of clinical and demographic characteristics according to CMF exposure pattern is presented in Table 3.

Among CMPA infants who underwent IgE testing, early CMF exposure within the first 72 h was not associated with a statistically significant difference in the distribution of IgE- and non-IgE-mediated phenotypes. IgE-mediated CMPA was observed in 8 of 32 infants (25%) with early CMF exposure and in 1 of 11 infants (9%) without early exposure (OR = 3.33; Fisher’s exact *p* ≈ 0.09). Non-IgE-mediated CMPA remained the predominant phenotype in both exposure groups.

## 4. Discussion

This study retrospectively investigated the association between exposure to CMF within the first three days of life and the subsequent development of CMPA, while also evaluating demographic, clinical, perinatal, and natal factors.

Among infants who received any CMF, 94% (76/81) had their first exposure within the first 72 h of life, whereas only 5 infants initiated CMF after this period. Notably, CMPA developed in 82% of infants who received CMF exclusively during the first 72 h and were subsequently breastfed, suggesting that early CMF exposure is a strong risk factor for disease development. Previous studies have supported this finding, although the timing and extent of exposure vary among reports. Studies comparing infants fed breast milk and amino acid-based formulas during the first three days with those receiving CMF have reported a significantly higher risk of CMPA in the latter group [18]. Therefore, our study focused specifically on CMF exposure within the first 72 h, a period characterized by minimal environmental impact on the microbiota and high immunological relevance. Nevertheless, because microbiota development continues for up to 100 days, this three-day window may be too narrow to capture some early microbial shifts [4].

The gut microbiome constitutes a dynamic ecosystem that exerts several beneficial functions for the host and plays a central role in immune regulation and the development of oral tolerance. Early-life dysbiosis has been shown to increase susceptibility to food allergens and may predispose infants to CMPA [19]. Although IgE-mediated CMPA is relatively less common, early CMF exposure within the first two weeks of life has been associated with increased risk [20]. Moreover, studies indicate that avoiding CMF during the first 72 h and delaying CMF introduction to a later period may reduce CMPA risk [18,21]. Therefore, the evaluation of CMF exposure specifically within the first 72 h was considered appropriate and clinically justified in the design of our study. For example, in a prospective study involving 1749 newborns, the prevalence of CMF use during the first three months was reported as 48%, and 57% of those later diagnosed with CMPA had received CMF [22]. Similarly, Stintzing et al. found that 64% of CMPA infants were exposed to CMF within the first week of life and 88% within the first month, rates significantly higher than those in the control group [23]. Although our study demonstrated an association between CMF exposure within the first 72 h of life and an increased risk of CMPA, several studies in the literature have reported different or even conflicting results regarding early exposure. For example, Díaz Martín et al. [24] published a consensus statement examining the impact of breastfeeding, hypoallergenic feeding, and early formula exposure during the first week of life on CMPA risk, and concluded that avoiding CMF in early life does not meaningfully reduce the likelihood of developing CMPA. Such discrepancies likely arise from methodological differences across studies, including variations in study design, population characteristics, the timing and amount of CMF exposure, breastfeeding practices, and diagnostic criteria. Therefore, while our findings support an association between early limited CMF exposure and CMPA development, they should be interpreted in the context of the broader literature, which contains heterogeneous and sometimes divergent perspectives. In a Taiwanese study, the incidence of CMPA was significantly lower among exclusively breastfed infants during the first four months compared with those receiving partial breastfeeding [25].

CMPA is characterized by sensitization, often polysensitization, to multiple milk proteins, particularly casein, β-lactoglobulin, and α-lactalbumin [26]. Clinical manifestations typically emerge during early infancy, usually following the introduction of cow’s milk-based formula [12]. The pathophysiology of CMPA is closely related to the immaturity of the neonatal immune and gastrointestinal systems. During early life, the intestinal barrier is functionally immature, and immune responses are predominantly Th2-driven, predisposing the infant to allergic sensitization [27].

Breast milk remains the optimal source of nutrition during the first six months of life and provides protection for gastrointestinal, immunological, and psychological development [28]. Exclusive breastfeeding shapes the gut microbiota and prevents premature exposure of the gastrointestinal tract to highly antigenic foods. It also supplies immunomodulatory components such as secretory IgA, transforming growth factor-β (TGF-β), and lactoferrin, which promote mucosal barrier maturation and the establishment of oral tolerance [27].

A large multicenter French study evaluating 1674 CMPA infants reported a mean age of 4.5 months (range 0.1–18 months) and a male predominance of 54% [29]. In our cohort, 51% of CMPA cases were male, and the mean age at diagnosis (corresponding to the initiation of the elimination diet) was 3.6 ± 1.9 months, consistent with previous reports showing a higher prevalence in early infancy.

CMPA can be classified as IgE-mediated, non-IgE-mediated, or mixed type [11]. In non-IgE-mediated CMPA, the most common gastrointestinal manifestations include bloody stools, constipation, vomiting, gastroesophageal reflux, delayed gastric emptying, diarrhea, perianal rash, poor appetite, and early satiety [30]. In our study, bloody and mucoid stools were the most frequent symptoms. Although early CMF exposure within the first 72 h increased the overall likelihood of developing CMPA, this exposure pattern did not meaningfully alter the distribution of IgE- versus non-IgE-mediated phenotypes. Non-IgE-mediated CMPA remained predominant regardless of early CMF exposure, consistent with the typical clinical presentation observed in early infancy.

Primary allergy-prevention studies classify infants with a positive family history of allergy in first-degree relatives (parents or siblings) as high-risk [31]. A multicenter Chinese study involving 6768 children identified 182 CMPA cases and found a significantly higher prevalence of family allergy history compared with controls [32]. Consistently, in our cohort, a positive family history of atopy was markedly more common in the CMPA group, underscoring the importance of familial predisposition in clinical risk assessment.

The association between cesarean delivery and CMPA remains controversial. Some studies have reported higher cesarean section rates and lower breastfeeding rates among CMPA patients [32]. In our study, cesarean delivery rates did not differ significantly between groups. However, CMF exposure within the first 72 h was more frequent among infants born by cesarean section, suggesting that cesarean delivery may act as an indirect risk factor by increasing early formula exposure rather than serving as a direct determinant of CMPA development.

Prematurity has also been discussed as a potential risk factor. In a study from Ireland including 144 preterm infants admitted to neonatal intensive care units, the prevalence of CMPA (1.4%) was comparable to that in the general population [33]. Similarly, another cohort study found no direct association between prematurity and CMPA [34]. Our findings were consistent with these observations, showing no significant difference in CMPA incidence by gestational age. Nonetheless, early CMF exposure within the first 72 h was more frequent among preterm infants, suggesting that the increased risk may derive from feeding practices rather than prematurity itself.

Previous studies have reported elevated eosinophil counts in CMPA patients compared with controls [35]. Interestingly, in our cohort, eosinophilia was more prevalent in the control group. This paradox may be explained by the smaller number of controls who underwent eosinophil testing and the predominance of non-IgE-mediated cases in our CMPA cohort. Additionally, the control infants were otherwise healthy, randomly selected during routine outpatient visits, and only a small subset had complete blood counts performed. Mild hypereosinophilia in this population often represents incidental, transient findings unrelated to allergic disease and may reflect recent viral infections or environmental factors rather than true atopic activity. These observations collectively indicate that eosinophil levels are not reliable diagnostic markers across all CMPA phenotypes and should be interpreted alongside clinical context, particularly in non-IgE-mediated disease.

The present study has several limitations. Complete blood count results were not available for all infants, and serum milk-specific IgE measurements could not be obtained in every case, which limited the ability to systematically and comprehensively evaluate sensitization patterns. In addition, detailed information regarding maternal and neonatal antibiotic exposure and the use of other medications could not be reliably extracted from medical records. Furthermore, aside from cases that received only a single CMF feeding followed by exclusive breastfeeding, precise quantitative data on the total number and volume of formula exposures were lacking, preventing a more detailed dose–response assessment.

Despite these limitations, the study also has important strengths. Focusing on a clearly defined, biologically critical period, the first 72 h of life provides a distinct temporal framework that has rarely been examined in previous research. Moreover, all CMPA diagnoses were established within the same tertiary-care center and strictly based on ESPGHAN-recommended elimination and oral food challenge procedures, ensuring diagnostic consistency and minimizing heterogeneity.

Larger, prospective multicenter studies are warranted to validate these findings and further clarify the temporal relationship between early CMF exposure and the development of CMPA.

## 5. Conclusions

In conclusion, CMF exposure during the first 72 h of life is a strong independent predictor of CMPA, although a family history of atopy remains the strongest risk factor. In addition to early CMF intake, cesarean delivery, prematurity, NICU admission, and a family history of atopy were all associated with increased CMPA risk in the multivariate analysis. These findings highlight the importance of minimizing unnecessary CMF supplementation during the immediate postnatal period and prioritizing early breastfeeding. Public health strategies that support breastfeeding initiation and reduce routine CMF use, especially in settings with high cesarean delivery rates, may help lower the overall incidence of CMPA.

## Figures and Tables

**Table 1:** Demographic and clinical characteristics of CMPA and control groups.

	CMPA (n = 106)	Control (n = 106)	*p* Value
Sex (Male, %)	54 (51)	55 (52)	0.500
Age (months, mean ± SD/median [min–max])	9.4 ± 6.7/8 (1–40)	12.0 ± 8.1/11 (2–37)	0.011
Age at diagnosis (months, mean ± SD/median [min–max])	3.6 ± 1.9/3 (1–12)	-	-
Family history of atopy, n (%)	63 (59)	21 (20)	<0.001
NICU admission, n (%)	26 (25)	28 (26)	0.437
Gestational age (Term/Preterm)	89 (84)/17 (16)	88 (83)/18 (17)	0.500
Mode of delivery (Cesarean/Vaginal)	81 (76)/25 (24)	73 (69)/33 (31)	0.140
Hypereosinophilia, n (%)	11 (12)	7 (29)	0.045
Milk-specific IgE (+), n (%)	9 (21)	-	-

CMPA, cow’s milk protein allergy; NICU, neonatal intensive care unit; IgE, immunoglobulin E; SD, standard deviation. *p* < 0.05 was considered statistically significant.

**Table 2:** Univariate and multivariate logistic regression analysis of risk factors for CMPA.

Variable	Univariate OR (95% CI)	*p* Value	Multivariate OR (95% CI)	*p* Value
Early CMF exposure (first 72 h)	2.21 (1.22–4.00)	0.009	2.35 (1.18–4.68)	0.016
Family history of atopy	5.93 (3.21–10.97)	<0.001	6.29 (3.31–11.92)	<0.001
Cesarean delivery	1.46 (0.80–2.69)	0.220	-	-
Prematurity	0.93 (0.45–1.93)	0.850	-	-
NICU admission	0.91 (0.49–1.68)	0.750	-	-

CMF, cow’s milk formula; CMPA: cow’s milk protein allergy NICU, neonatal intensive care unit; OR, odds ratio; CI, confidence interval. *p* < 0.05 was considered statistically significant.

**Table 3:** Comparison of clinical and demographic characteristics according to the pattern of CMF exposure, including early limited CMF intake confined to the first 72 hours, followed by exclusive breastfeeding.

Variable	Early Limited CMF Exposure (First 72 h Only) n (%)	Continued CMF Exposure Beyond 72 h n (%)	*p* Value
CMPA cases	40 (46)	46 (54)	<0.001
Controls	9 (12)	65 (88)	-
Cesarean/Vaginal	38 (30)/11 (31)	87 (70)/24 (69)	0.907
Female/Male	22 (29)/27 (31)	52 (71)/59 (69)	0.820
Term/Preterm	40 (32)/9 (26)	86 (68)/25 (74)	0.554
NICU admission	14 (28)	36 (72)	0.627
Family history of atopy	25 (40)	38 (60)	0.119

CMF, cow’s milk formula; CMPA: cow’s milk protein allergy NICU, neonatal intensive care unit. *p* < 0.05 was considered statistically significant. Dipnot: All infants in this table had CMF exposure; the comparison reflects different exposure patterns rather than the presence or absence of CMF exposure.

## Data Availability

The data that support the findings of this study are available from the corresponding author upon reasonable request. Due to ethical and privacy restrictions, the dataset is not publicly available.

## Abbreviations

The following abbreviations are used in this manuscript:

CMPA	Cow’s Milk Protein Allergy
CMF	Cow’s Milk Formula
HMF	Human Milk Fortifiers
IgE	Immunoglobulin E
NICU	Neonatal Intensive Care Unit
WHO	World Health Organization
